# Incomplete recovery of mechanical and endocrine left atrial functions one month after electrical cardioversion for persistent atrial fibrillation: a pilot study

**DOI:** 10.1186/1479-5876-12-51

**Published:** 2014-02-22

**Authors:** Anne Bernard-Brunet, Christophe Saint Etienne, Eric Piver, Noura Zannad, Jean-Christophe Pagés, Laurent Fauchier, Dominique Babuty

**Affiliations:** 1 Cardiology Department, François Rabelais University, 37020 Tours, France; 2 Biochemistry Department, François Rabelais University, 37020 Tours, France

**Keywords:** Atrial fibrillation, Electrical cardioversion, Left atrial remodeling, Speckle-tracking echocardiography, Atrial natriuretic peptide

## Abstract

**Background:**

Restoration of the mechanical and endocrine functions of the left atrium remains controversial after electrical cardioversion treatment for persistent atrial fibrillation. The objective of the prospective study was to describe the recovery of the endocrine and mechanical functions of the left atrium.

**Methods:**

Evaluation of left atrium recovery after electrical cardioversion by the new speckle-tracking echocardiography technique and proANP measurement.

**Results:**

Twenty patients suffering from persistent atrial fibrillation with no alteration of left ventricular ejection fraction were prospectively evaluated at baseline and then one month later by echocardiography, measuring left atrial volume and left atrial deformation (MPALS), as well as the proANP and BNP concentrations. One month after cardioversion 10 patients remained in sinus rhythm and 10 showed recurrent atrial fibrillation. No significant differences between the two groups in terms of clinical, echocardiographic and endocrine parameters were observed at baseline evaluation. We observed a significant reduction of left atrial volume only in the sinus group, whereas restoration of the left atrial deformation was only partial (18%) in that group. By contrast, we registered no significant changes in ANP concentration at one month in either the sinus or the atrial fibrillation groups.

**Conclusion:**

These results suggest that restoration of left atrium mechanical function is only partial one month after treatment of persistent atrial fibrillation by electrical cardioversion, whereas a significant reduction of left atrial volume was noted, explaining the remaining high level of ANP in the sinus group.

## Background

Atrial fibrillation (AF) is the most frequently encountered arrhythmia, affecting 2% of the population [1]. Its frequency is on the increase, in parallel with the aging of the population, making the management of AF with antithrombotic drugs or antiarrhythmic therapy part of daily medical practice. AF is associated with increased rates of mortality and morbidity due to heart failure, stroke and other thromboembolic events as well as a reduced capacity for exercise and reduced quality of life [2]. AF also leads to left ventricular dysfunction [2] and a loss of mechanical function of the left atrium [3,4]. Whether paroxysmal, persistent or permanent, AF also stimulates the secretion of a member of the natriuretic peptide family – atrial natriuretic peptide (ANP) [3-5]. The natriuretic peptide family includes, in addition to ANP, two other members: brain natriuretic peptide (BNP) and C-type natriuretic peptide (CNP). ANP and BNP are mainly produced by atrial and ventricular cardiomyocytes, respectively. Atrial cardiomyocytes store prohormones or propeptides, such as proANP, in secretory granules. The main mechanical stimulus triggering the secretion of ANP and BNP is considered to be the distension of atrial and ventricular cardiomyocytes [5-8]. The mechanical and endocrinal effects linked to the restoration of the sinus rhythm (SR) by cardioversion or radiofrequency ablation remain controversial. These discrepancies can probably be explained by the techniques used to evaluate the mechanical function of the LA [4,9-13]. Most of the studies cited above have merely provided a rough estimation of LA mechanical function by measuring the size and volume of the LA. However, over the last few years, more advanced techniques to evaluate the mechanical function of the LA have been developed, for example by analyzing the myocardial deformation (or strain) of the atrial wall by tissue Doppler imaging (TDI), and more recently, by an angle-independent technique: speckle-tracking echocardiography [14].

We hypothetized that left atrium mechanical function (distensibility) was incomplete one month after the restoration of sinus rhythm, explaining a prolonged high level of ANP. We carried out a prospective study to evaluate the LA mechanical function by the new angle-independent and reproducible speckle-tracking echocardiography technique, and to gauge the endocrine function by measuring the levels of proANP and BNP at baseline and one month after cardioversion.

## Methods

### Study design

Twenty-nine patients suffering from symptomatic non-valvular persistent AF who were admitted for direct current cardioversion were prospectively recruited. All patients referred to our center for electrical cardioversion received an adequate anticoagulant (VK antagonist with an INR between 2 and 3 during at least one month). Persistent AF was defined as AF lasting for at least 7 days but less than one year, and requiring direct current cardioversion [2]. Exclusion criteria for the study were a left ventricular ejection fraction (LVEF) below 45%, contraindication to electrical cardioversion (intracardiac thrombus, international normalized ratio, INR <2, contraindication to anesthesia), refusal of consent, and age <18 years. Only symptomatic patients were included in the study in order to evaluate the recurrence of atrial fibrillation after electrical cardioversion. The study protocol was approved by the local ethics committee (CCP Tours Région Centre Ouest 1) for human research in Tours. All patients signed informed consent forms.

Patients were evaluated at baseline (one day before cardioversion) and one month later, as follows: Physical examination and standard 12-lead electrocardiogram (ECG); standard transthoracic echocardiography with a Vivid I digital ultrasound system (GE Medical Systems Ultrasound) and digital cineloop format for off-line analysis using a dedicated software package (EchoPac PC, version 110.1.2; GE Healthcare); clinically required blood samples were collected, in addition to a sample for the evaluation of natriuretic peptides (BNP and proANP). LA function was reevaluated one month after cardioversion because that is generally the estimated time necessary for recovery from atrial stunning or left atrial dysfunction [2]. Recurrences of atrial fibrillation after electrical cardioversion were diagnosed on the basis of symptoms, resting ECG and 24-hour ambulatory ECG performed by the generalist practitioner or the cardiologist.

Direct current cardioversion was performed under general anesthesia, with one to three biphasic shocks of increasing energy, from 100 to 200 J. Patients were followed up one month later with a physical examination, ECG, echocardiography and blood samples for ANP, BNP measurement.

### Echocardiographic measurements

**Figure 1:**
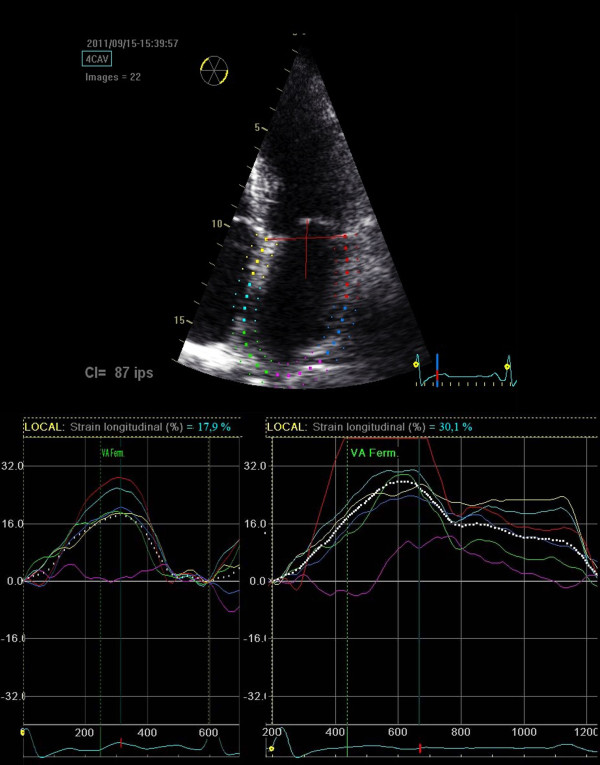
**Left atrial speckle-tracking echocardiography.** Top. Evaluation of atrial deformation by the speckle-tracking technique. The endocardial contour of the LA is traced in a 4-chamber view. The thickness of the region of interest is then defined. The software automatically tracks atrial wall movements, allowing fine adjustments and verification in real time. Bottom. Strain curves for the same patient as in top figure, examined during AF (left) and after 1 month of SR (right). Each curve represents one of the six LA wall segments; the dotted line indicates the mean strain.

All patients were examined in the left lateral decubitus position by M-mode, two-dimensional (2D), Doppler and TDI echocardiography, using a 3.5-MHz transducer (GE Medical Systems). Standard M-mode and 2D images were obtained from 3 consecutive breath-hold cardiac cycles and were stored in cineloop format. Apical four- and two- chamber-view images were obtained using conventional two-dimensional gray scale echocardiography, during breath hold with a stable ECG recording. Particular attention was given to obtaining an adequate gray scale image, allowing reliable delineation of myocardial tissue and extracardiac structures. LA volume was calculated according to the formula 8/3π[(LA area in apical 4-chamber view * LA area in apical 2-chamber view)/LA length. LA volume was indexed to body surface area. The anteroposterior diameter of the LA was also measured from the parasternal long-axis view [15]. The left ventricular (LV) end-diastolic diameter (LVEDD) was obtained from M-mode images of the parasternal long-axis view. LV end-diastolic and end-systolic volumes were measured from apical 2- and 4-chamber views, and LVEF was calculated using Simpson’s rule [15]. The LV volumes and LVEDD were also indexed to the body surface area. Speckle-tracking echocardiography is a recent non-Doppler method to evaluate strain from standard two-dimensional grayscale cineloops [16]. By tracing the endocardial contour in both 4- and 2-chamber views of an end-systolic frame using a point-and-click approach, the software automatically tracks the atrial wall in subsequent frames. Tracking accuracy can be verified and corrected in real time by adjusting the region of interest (Figure 1). We used the highest frame rate available (60–80 frames per second).

Off-line analysis was then performed as follows. We measured the deformation of six segments located along the septum, the roof and the lateral wall of the LA cavity as seen in a 4-chamber apical view, and repeated the process with a 2-chamber view. A curve representing the average of the strain curves of the six segments was automatically plotted (Figure 1), where the maximum value or “peak strain” indicated the maximum global deformation of the LA. This value is positive, as it represents the lengthening of the LA wall during LV systole due to the filling of the atrial cavity with blood from the pulmonary veins while the mitral valve is closed. These measurements were repeated for three different cardiac cycles to calculate the mean 4-chamber peak atrial longitudinal strain (4C-PALS) and the mean 2-chamber PALS (2C-PALS). Finally, the mean PALS (MPALS), was calculated from both 4C-PALS and 2C-PALS [16]. Mitral regurgitation was classified as mild, moderate or severe, according to current guidelines [17].

Two independent physicians analyzed the echocardiographic measurements on ten selected subjects in order to define the intra- and inter-observer variability.

### Biological assays

Because proANP half-life is longer than that of ANP, as measured ProANP concentrations in the serum, we used the MR-proANP kit (BRAHMS, Clichy, France). This sandwich immunoassay uses an antibody against the mid-region of proANP (amino acids 53–90). In 325 healthy individuals, proANP concentrations ranged from 9.6 to 313 pmol/L. The median was 45 pmol/L (95% confidence interval: 43.0–49.1 pmol/L). The 99th percentile for the control population was 197.5 pmol/L; there was no significant difference in the range or median of proANP values between males and females (mean: 45.3 pmol/L in males, 45.0 pmol/L in females) [18].

BNP levels were assayed in EDTA plasma. As for proANP, we used a sandwich immunoassay: the Triage® BNP Test (Biosite Diagnostics, Vélizy-Villacoublay, France) for the Access 2 system (Beckman Coulter Immunoassay Systems, Villepinte, France). According to manufacturer claims, in 1286 subjects without chronic heart failure, the median value was 12.3 pg/mL and the 95th percentile was 73.5 pg/mL.

### Statistical analysis

Data were expressed as means ± standard deviations. Since normality testing of the different variables showed a non-Gaussian distribution, non-parametric statistical tests were applied. Differences in the means of the various parameters before and after cardioversion were assessed by a Wilcoxon test for paired series. Differences in the means of baseline parameters between patients with AF and patients in SR one month after cardioversion were assessed by a Wilcoxon test for unpaired series. Correlations between LA volume or MPALS and ANP or BNP concentrations were evaluated by the Spearman correlation coefficient. A p-value < 0.05 was considered to be significant. Statistical analyses were performed using JMP 9.0.1 software (SAS Institute, Cary, NC, USA). For the reproducibility of the parameters, results were analyzed using Bland-Altman comparison and coefficient of variability.

Based on our results obtained at baseline and one month after electrical cardioversion in a limited number of patients, we calculated the theoretical number of patients it should be necessary to recruit to reach a significant difference between the two groups in term of ANP level (website: http://www.spc.univ-lyon1.fr/mfcalc:NSN/crit%20continu.htm).

## Results

### Study population

The study population initially consisted of 29 consecutive patients with persistent AF who were addressed for cardioversion. Nine patients were excluded from the study because cardioversion failed or because the patients were lost to one-month follow-up. The clinical characteristics of these nine excluded patients did not differ from other patients in terms of age (65.3 ± 10 years), sex (40% female), associated cardiac disease, ANP level (248.9 ± 108 pmol/l), BNP level (240 ± 111 pg/ml) or echocardiographic parameters (LA volume 45.2 ml/m^2^, 4C-PALS mean 11.8%; 2C-PALS mean 9%; MAPS mean 10.6%). The main characteristics of the remaining 20 patients are summarized in Table 1. None of the patients had significant mitral regurgitation. One month after cardioversion, 10 patients displayed SR (SR group) while 10 others displayed a recurrence of AF (AF group). In the AF group all patients had at least one symptomatic episode of AF documented on ECG and were in AF on the day of the second evaluation. All the SR patients were asymptomatic with normal sinus rhythm on resting ECG and 24-hour ambulatory ECG. Clinical and echocardiographic characteristics were not significantly different at baseline between the SR and AF groups (Table 1).

**Table 1:** Patients’ baseline characteristics

**Mean values at baseline**	**SR group (n = 10)**	**AF group (n = 10)**	**P value**
**Age (years)**	68.2 ± 10.6	66.9 ± 11.0	NS
**Sex ratio n, (% female)**	10 (50)	10 (50)	NS
**Average time between the diagnosis of AF and cardioversion (weeks)**	11.6 ± 7.7	14.1 ± 10.2	NS
**Cardiac disease n, (%)**			NS
**Coronary disease**	1(10)	1(10)	
**Hypertension**	5 (50)	3 (33.3)	
**Diabetes mellitus**	1 (10)	1 (10)	
**Antiarrhthymic drugs**			NS
**Amiodarone**	**7**	8	
**Flecainide**	**1**	1	
**Sotalol**	**2**	1	
**Beta blockers**	**6**	6	

### Echocardiographic parameters

Although great care was taken to ensure the quality of the data collected, we excluded one 4-chamber view and three 2-chamber views because of poor quality. Inter-observer variability coefficients of 4C-PALS, 2C-PALS and MPALS were 9%, 13% and 8%, respectively. Intra-observer variability coefficients of 4C-PALS, 2C-PALS and global MPALS were 5%, 8% and 6%, respectively. No significant difference was observed between the two independent physicians who analyzed the echocardiographic recordings. Among the standard echocardiographic parameters measured, the mean LA volume did not differ between the SR and AF groups at baseline (Table 2). Nonetheless, a significant diminution of LA volume was observed in the SR group between baseline and the one-month time point (42.3 ± 7.4 vs. 36 ± 4.4 mL/m^2^, p < 0.01), whereas the mean LA volume of patients in the AF group remained unchanged at that point (47 ± 15.7 vs. 47.5 ± 18.4 mL/m^2^, p = 0.81). We noted a slight improvement in LVEF in the SR group one month after cardioversion (54 ± 11.1 vs. 60.4 ± 5.6%, p = 0.04), while no improvement in LVEF was seen in the AF group (50.5 ± 7.9 vs. 52.9 ± 11.6%, p = 0.77).

**Table 2:** Endocrine and echocardiographic parameters at baseline evaluation

**Mean values at baseline**	**SR group (n = 10)**	**AF group (n = 10)**	**p**
**proANP (pmol/L)**	235.6 ± 62.8	226.9 ± 151.4	NS
**BNP (pg/mL)**	205.9 ± 99.0	197 ± 139.0	NS
**LA volume/body surface area (ml/m**^**2**^**)**	42.3 ± 7.4	47.0 ± 15.7	NS
**LVEF (%)**	54.0 ± 11.1	50.5 ± 7.9	NS
**4C-PALS (%)**	10.9 ± 4.2	10.3 ± 4.7	NS
**2C-PALS (%)**	9.8 ± 5.1	11.8 ± 3.8	NS
**MPALS (%)**	10.4 ± 4.4	10.4 ± 4.4	NS

**Figure 2:**
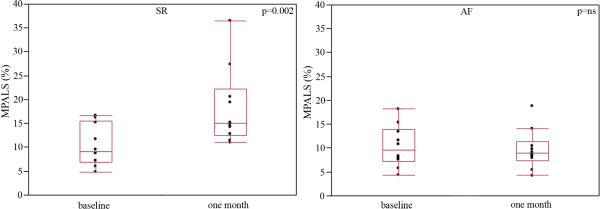
**Variation of mean peak atrial longitudinal strain after cardioversion.** MPALS (%) at baseline and one month after cardioversion in the SR (left) and AF(right) groups. Results are expressed as box-and-whisker plots. The lines inside the boxes indicate the median (50th percentile), the limits of the boxes indicate the 25th and 75th percentiles, and the whiskers indicate the 10th and 90th percentiles.

Not surprisingly, an analysis of atrial myocardial deformation revealed no difference in MPALS at baseline between the SR and AF groups (Table 2). One month after cardioversion, MPALS increased significantly by 76.9% in the SR group, whereas no significant change was observed in the AF group (Figure 2). Finally, the variation in the MPALS of all patients studied between baseline and the one-month follow-up was well correlated with the variation in LA volume (Spearman ρ = −0.71, p < 0.001).

### Biological parameters

**Figure 3:**
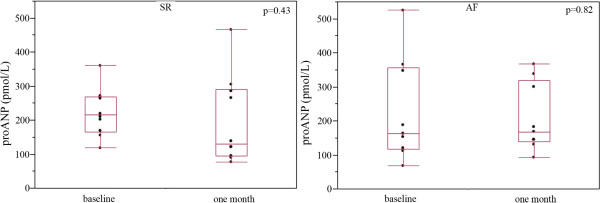
**Variation of proANP concentrations after cardioversion.** ProANP concentrations (pmol/L) at baseline and one month after cardioversion in the SR (left) and AF (right) groups. Results are expressed as box-and-whisker plots. The lines inside the boxes indicate the median (50th percentile), the limits of the boxes indicate the 25th and 75th percentiles, and the whiskers indicate the 10th and 90th percentiles.

High levels of proANP were observed in all patients (Table 2), but BNP was not greatly increased in either group. No difference between the SR and AF groups was observed for proANP or BNP concentrations at baseline (Table 2). ProANP concentrations one month after cardioversion were not significantly different between the SR (207 ± 130.4 pmol/l) and AF groups (207 ± 100.1 pmol/l). In the SR group we observed a slight decrease in ANP level without reaching statistical significance (Figure 3). In the AF group the evolution of ANP level was similar that of the other group at one month (Figure 3). Likewise, we found no significant difference between BNP concentrations at baseline and those at one month in either the SR group (205.9 ± 99 vs. 182.2 ± 180.4 pg/mL respectively, p = 0.30) or the AF group (197 ± 139 vs. 182.5 ± 107.1 pg/mL respectively, p = 0.91).

Based on the variation of ANP levels between baseline and one-month evaluations and the difference of mean ANP level in the two groups observed in our pilot study, it would be necessary to recruit 976 patients in order to reach a statistical significant difference that could differentiate the two groups.

### Correlation between mechanical function and endocrine function

**Figure 4:**
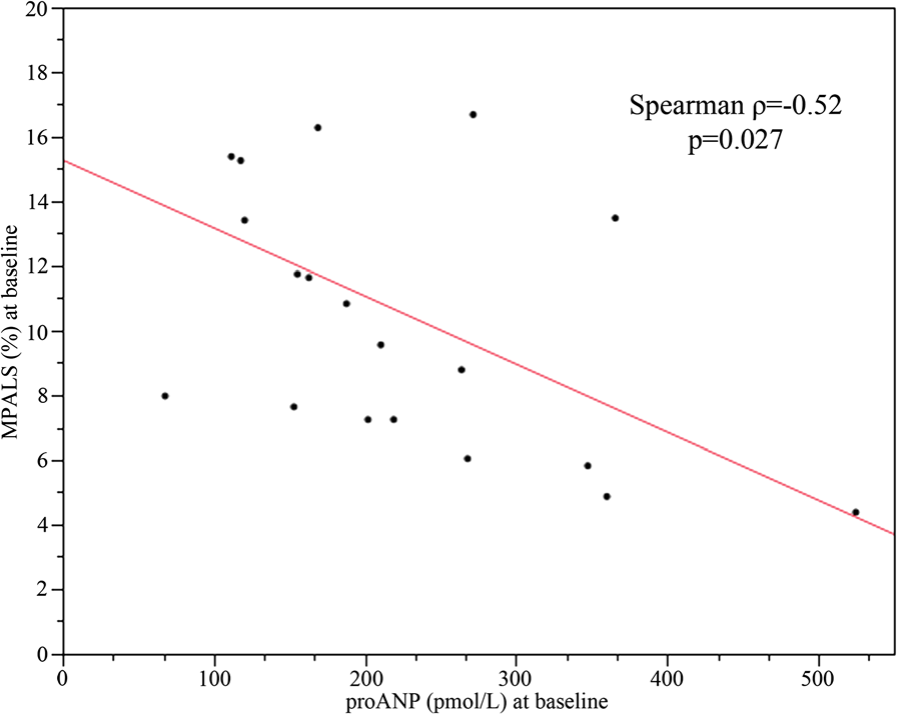
**Correlation between MPALS and proANP concentration at baseline.** Correlation between MPALS (%) and proANP concentrations (pmol/L) at baseline for all patients studied (SR and AF groups) (Spearman ρ = −0.52, p = 0.027).

Before cardioversion, proANP concentrations were negatively correlated with MPALS (Spearman ρ = −0.52, p = 0.027; Figure 4) but not correlated with LA volume (Spearman ρ = 0.31, p = 0.21). In contrast, there was no correlation between BNP concentrations and MPALS at baseline (Spearman ρ = −0.32, p = 0.14) or between BNP concentrations and LA volume at baseline (Spearman ρ = 0.33, p = 0.15).

One month after cardioversion, proANP concentrations were no longer correlated with either MPALS (Spearman ρ = −0.34, p = 0.2) or LA volume (Spearman ρ = 0.32, p = 0.22). There was no correlation of BNP concentrations with MPALS or LA volume at one month (Spearman ρ = −0.29, p = 0.25 and Spearman ρ = 0.13, respectively, p = 0.6).

## Discussion

Our study shows that in patients with persistent AF treated by cardioversion, LA mechanical function recovers faster than endocrine function, but that LA distensibility is only partial one month after sinus rhythm has been restored. To our knowledge, this is the first study assessing simultaneously the relationship between LA mechanical function, as evaluated by speckle-tracking echocardiography, and variations in proANP levels one month after cardioversion. Previous studies evaluated either the mechanical LA function or the endocrine function separately [13,19], but the relationship between the two functions could not be established and discussed.

Our population is quite similar in mean age, sex ratio and duration of AF to those in previous studies evaluating strain among patients with AF [19-21]. Furthermore, there is no difference in these parameters between the SR and AF groups at baseline. The rate of recurrence of AF at one month despite successful cardioversion is 50% in our study, which is comparable to that of other studies [22,23].

### Speckle-tracking echocardiography and LA mechanical function

Over the last few years new echocardiographic techniques have been developed and used to analyze myocardial deformation non-invasively. Di Salvo *et al*. [20] have used Tissue Doppler Imaging to study LA myocardial deformation in patients with a history of persistent AF. They show that the atrial lengthening (represented by a positive peak in strain) that occurs during ventricular ejection is significantly reduced in patients with AF. Our study has associated standard echocardiographic measurements of improved cardiac performance during SR, such as an increase in LVEF and a reduction in LA volume, with novel parameters such as an improvement in MPALS. Since we observe a negative correlation between the variation in LA volume and the variation in MPALS, we may presume that LA remodeling is a key mechanism underlying LA functional improvement. Dell’Era *et al*. [19] have reported this after using the new angle-independent technique of speckle-tracking echocardiography in a larger study including 73 patients. They reported a similar increase in MPALS (11.4 ± 5.2% in AF and 17.2 ± 7.5% after one month in SR), using the speckle-tracking technique. However, the recovery of mechanical function one month after successful cardioversion appears to be only partial, as MPALS in both our study and the Dell’Era study [19] (around 18% in both) is considerably lower than that of 60 healthy individuals in the report by Cameli *et al*. [16] (42.2 ± 6.1%; 5–95 percentile range: 32.2–53.2%). Our results demonstrate that atrial stunning lasts longer than one month after cardioversion. It would therefore be of interest to assess MPALS at longer intervals after cardioversion in order to determine the time to complete recovery.

### Natriuretic peptides and LA endocrine function

The particularity of our study is its focus on the link between the mechanical and endocrine functions of the LA. Indeed, our findings indicate that baseline proANP concentrations are negatively correlated with baseline MPALS, but no correlation is observed between proANP concentrations and LA volume. After one month in SR, the correlation between proANP levels and MPALS disappears, because mechanical function tends to improve, whereas proANP concentrations remain the same. Wozakowska-Kaplon has shown that mean ANP and BNP levels decrease 24 hours after electric shock and successful cardioversion [24], and that this is followed by stabilization of the ANP level or a gradual increase in ANP levels over the next 30 days [24,25]. Our results confirmed this previous observation. Other authors have reported a decrease in ANP concentrations after cardioversion [11,26], but the interval between cardioversion and the second ANP measurement is highly variable (1 to 180 days). Experimental and clinical studies suggest that atrial volume, pressure, and wall stretch are the main determinants of ANP secretion [5,6,11], although the precise identity of the trigger is still under discussion. Since LA volume decreases after AF cardioversion, we could expect a decrease in ANP concentrations. However, the lack of any such findings can be explained by the results of our echocardiographic analysis. Indeed, using the speckle-tracking technique, we have demonstrated that one month after cardioversion, the distensibility (MPALS) of the LA is not fully restored, explaining the persistently high levels of ANP secretion.

These preliminary results suggest performing an evaluation of the mechanical and endocrinal functions of LA two or three months after cardioversion in selected patients. This point could be important for patients with a low CHA_2_DS_2_-VASC score who do not need permanent anticoagulation. In absence of complete restoration of the mechanical and endocrine functions of LA one month after electrical cardioversion a prolongation of anti-coagulant agents could be proposed to the patients. Several echocardiographic studies have established in AF patients a close relationship between the risk of thrombo embolism and the LA mechanical function [2]. Speckle-tracking echocardiography is a recent echocardiographic technique that can accurately explore the contractility and the distensibility of LA. Its diffusion and development could be a useful new tool, associated with the measurement of ANP level, for the analysis of LA.

## Conclusions

Our pilot study shows that one month after cardioversion for AF, neither the mechanical function of the LA, especially distensibility as quantified by speckle-tracking echocardiography, nor its endocrine function are fully restored in SR patients, demonstrating that atrial stunning can exceed one month. The incomplete recovery of LA mechanical function contributes to high ANP concentration that favors a prothrombotic state. The fact that complete recovery time of endocrinal and mechanical functions of the left atrium after electrical cardioversion is longer than one month suggests that selected patients should be reexamined two or three months later before changing therapeutic agents, especially anticoagulants. A further expanded study would be necessary to confirm these results and their potential therapeutic applications.

## Abbreviations

ANP: Atrial natriuretic peptide; BNP: Brain natriuretic peptide; CNP: C-type natriuretic peptide; AF: Atrial fibrillation; TDI: Tissue doppler imaging; LA: Left atrium; MPALS: Left atrial deformation; LVEDD: Left ventricular end-diastolic diameter; LVEF: Left ventricular ejection fraction; SR: Sinus rhythm; 2C-PALS: 2-chamber peak atrial longitudinal strain; 4C-PALS: 4-chamber peak atrial longitudinal strain; MPALS: Mean peak atrial longitudinal strain.

## Competing interests

The authors declare that they have no competing interests.

## Authors’ contributions

Study concept: ABB, EP, NZ, DB. Echocardiography: ABB, CSE, NZ. Laboratory investigations: EP, JCP. Statistics: CSE, LF, DB. Manuscript drafting: ABB, CSE, LF, DB. All authors read and approved the final manuscript.
